# A case report of delayed treatment of acute exertional osteofascial compartment syndrome in the anterior compartment of the calf

**DOI:** 10.1097/MD.0000000000032449

**Published:** 2022-12-30

**Authors:** Shiwei Liu, Congcong Wang, Wenjing Song, Jun Wang, Shibo Zhao

**Affiliations:** a Joint Surgery Department, the First Affiliated Hospital of Weifang Medical University, Weifang, Shandong, China; b Oncology Department, the First Affiliated Hospital of Weifang Medical University, Weifang, Shandong, China.

**Keywords:** acute exertional osteofascial compartment syndrome, fasciotomy, nerve injury, vacuum sealing drainage

## Abstract

Acute exertional osteofascial compartment syndrome (OCS) is a rare cause of lower-leg pain and is often associated with delayed diagnosis, which can lead to irreversible muscle and nerve damage.

**Patient concerns:**

A 23-year-old man presented with acute-onset anterior calf pain and ankle dorsiflexion after hiking.

**Diagnosis:**

The patient’s pain was initially diagnosed as muscle strain at a county hospital, but was eventually diagnosed as OCS at our hospital 8 days after the injury. This case presents several challenges in the diagnosis and treatment phases.

**Interventions:**

Three surgeries were performed in total. On the day after admission (9 days after injury), fasciotomy was performed, followed by vacuum sealing drainage (VSD). Six days after the first surgery, necrotic muscle debridement was performed and VSD was reperformed. Ten days after the second surgery, the covering foam material was removed and the incision was sutured.

**Outcomes:**

Satisfactory postoperative results were achieved. The erythrocyte sedimentation rate, C-reactive protein level, and white blood cell count were within normal ranges. The skin healed well, and nerve damage and muscle strength improved significantly 3 months after surgery.

**Lessons:**

OCS in the absence of trauma or fracture is rare, but treatment delays can have devastating consequences. Acute nontraumatic OCS requires prompt diagnosis and surgical intervention to prevent adverse outcomes. VSD is an effective surgical treatment for this disease.

## 1. Introduction

Acute exertional osteofascial compartment syndrome (OCS) is characterized by a rise in pressure within a closed fascial space in the absence of a specific traumatic event.^[1]^ This rise in pressure can impair the circulation and function of the tissues within that space, leading to ischemia and tissue dysfunction.^[2,3]^ Although poorly understood, acute exertional OCS typically occurs in high-level athletes or after a period of strenuous exercise.^[4]^ After the diagnosis of OCS, decompression fasciotomy should be performed immediately to prevent this condition from leading to gangrene, crush ischemic muscle spasm, liver and kidney function damage, and other life-threatening syndromes.^[5]^ Correct early diagnosis of OCS is very important, because a diagnostic delay is the most common cause of treatment failure.

Herein, we report a case in which decompression fasciotomy was performed 8 days after the onset of acute exertional OCS. The patient was initially diagnosed with muscle strain at a county hospital and treated with ice compresses, hot compresses, and oral nonsteroidal anti-inflammatory drugs. However, the pain gradually worsened and gross hematuria was observed. Eight days later, the patient presented to our hospital and was admitted for emergency surgery. Three months after surgery, the patient recovered well. We believe that by reporting the clinical characteristics, detailed diagnosis, and treatment of the patient, combined with a review of the literature, we can help provide more early correct diagnoses and further explore treatment options for acute exertional OCS.

## 2. Case presentation

**Figure 1.**
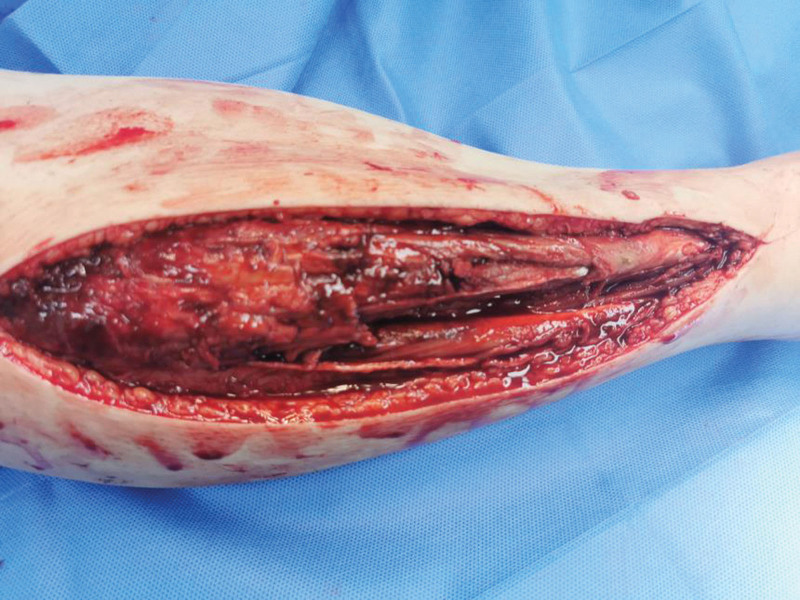
Appearance at the time of the first surgery.

**Figure 2.**
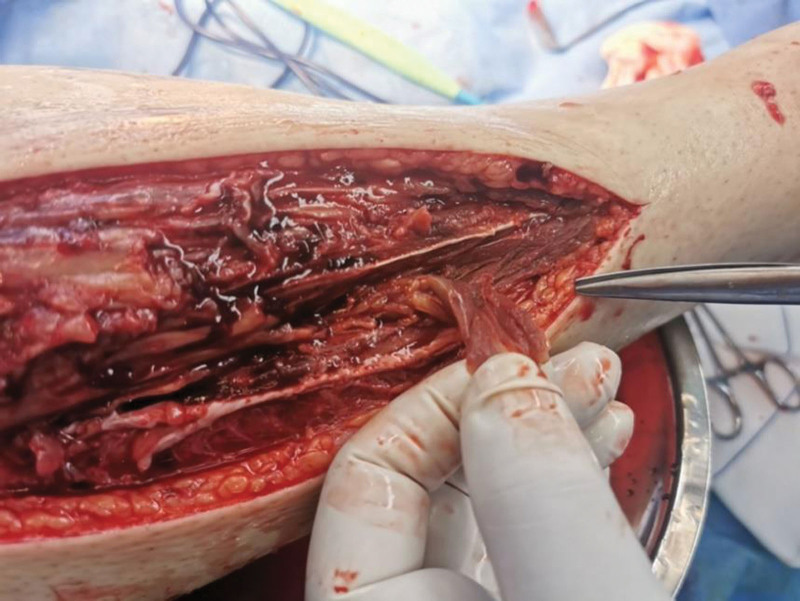
Appearance at the time of the first surgery.

**Figure 3.**
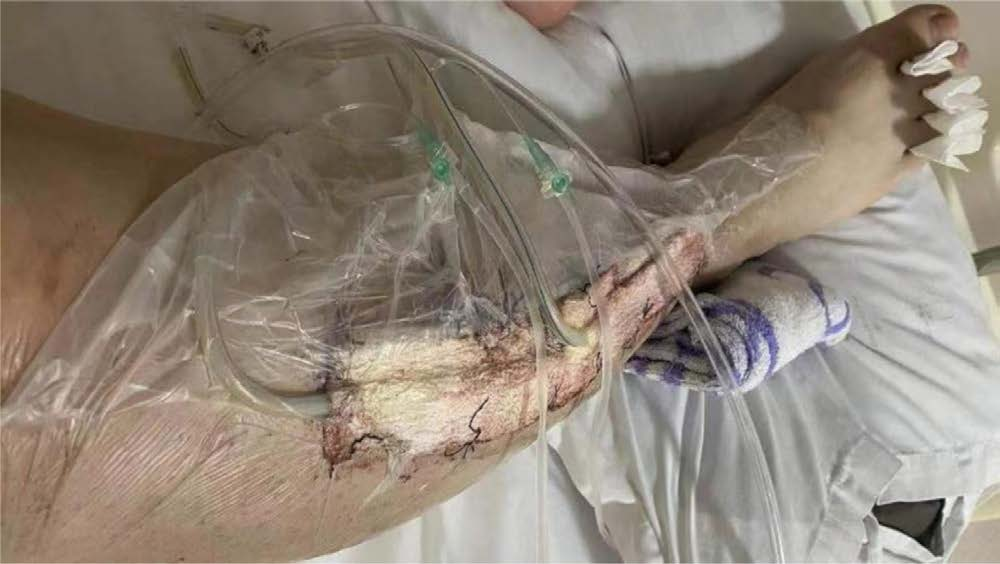
External phase of VSD closure covering the wound after the first surgery.

**Figure 4.**
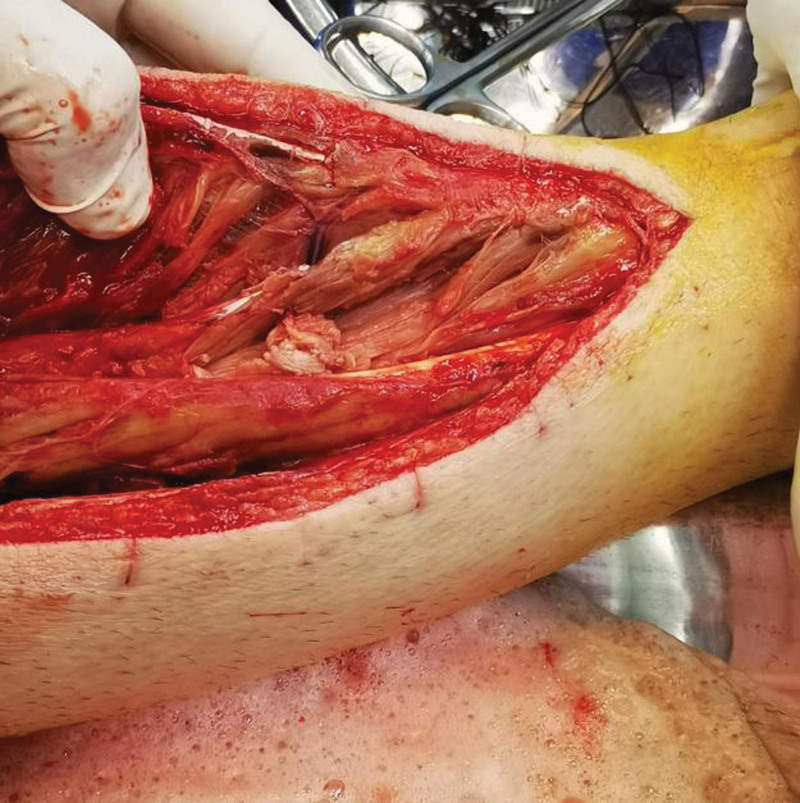
Appearance at the time of the second surgery.

**Figure 5.**
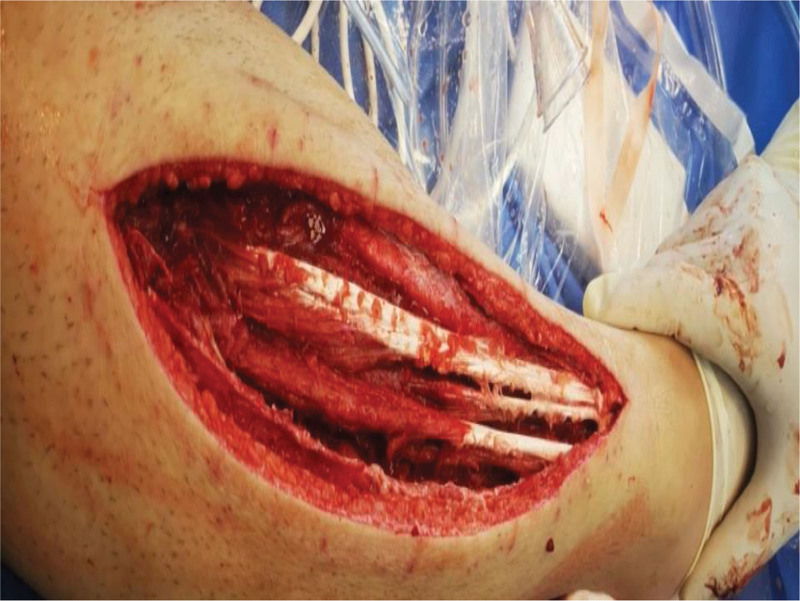
External phase after the second surgery to clean up necrotic muscle.

**Figure 6.**
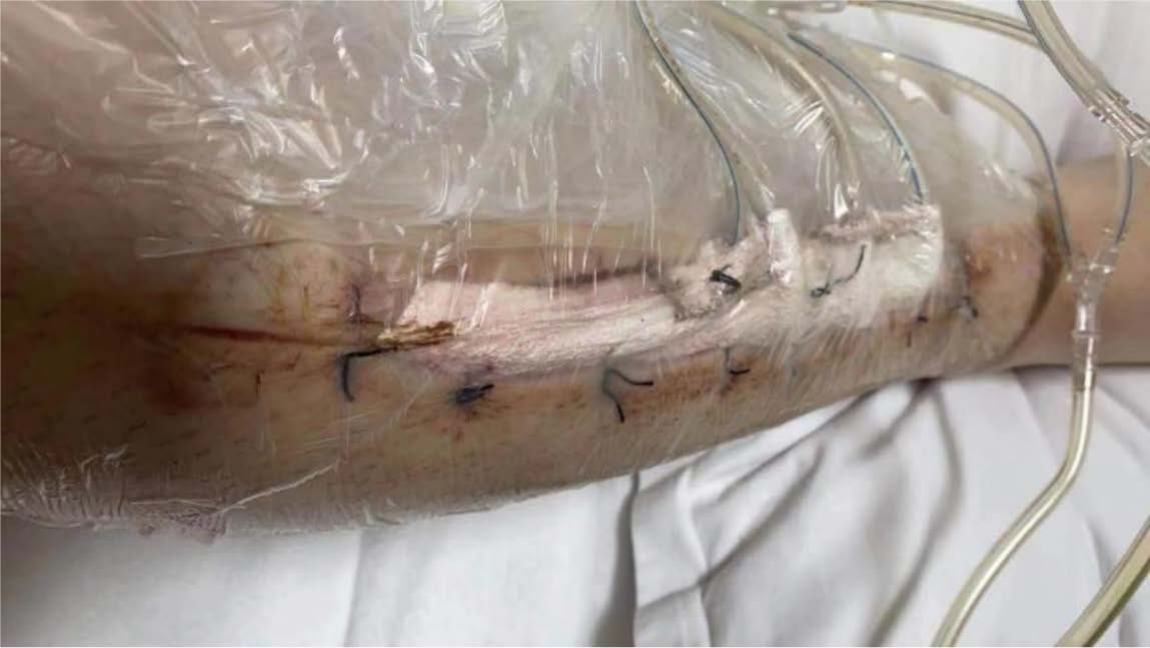
External phase of VSD closure covering the wound after the second surgery.

**Figure 7.**
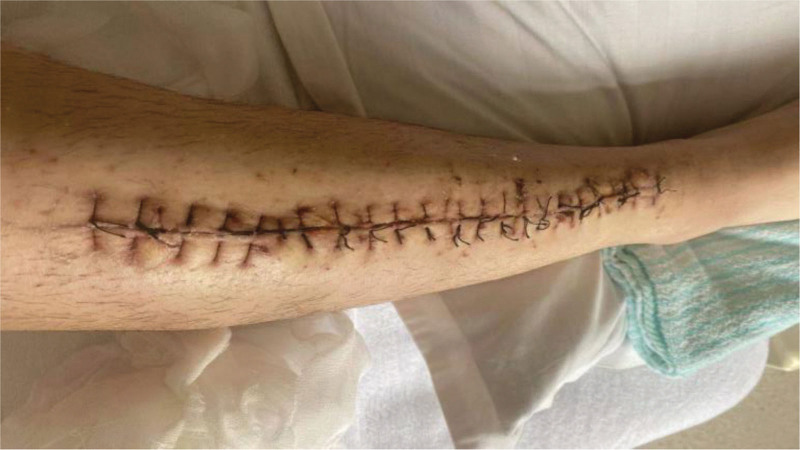
External phase of the third surgical incision suture.

**Figure 8.**
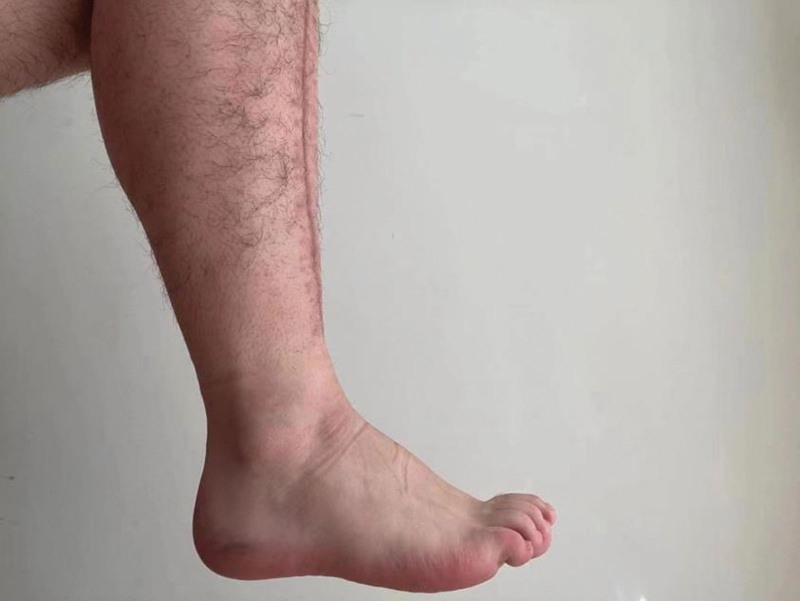
Image of ankle dorsiflexion of the patient 3 months after discharge from the hospital.

**Figure 9.**
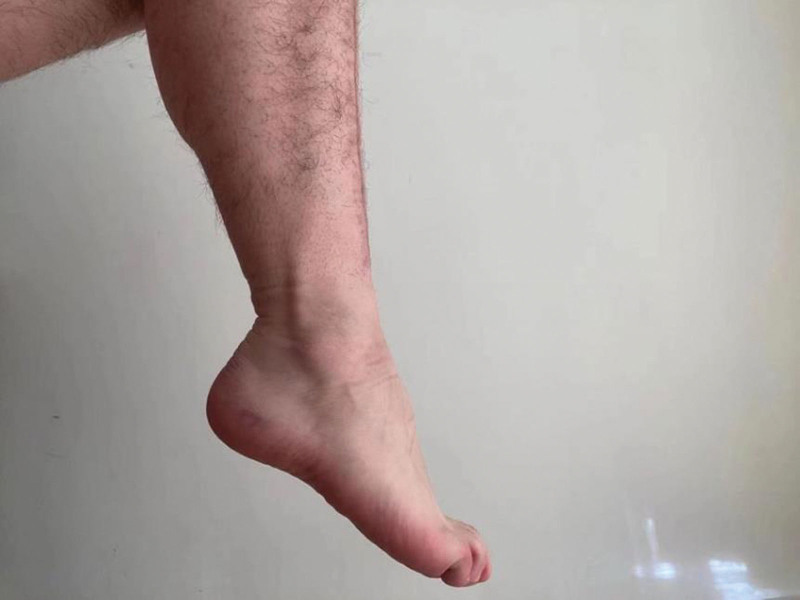
Image of ankle plantarflexion of the patient 3 months after discharge from the hospital.

The patient, a 23-year-old male, was admitted to our hospital on October 4, 2020, due to “right calf pain and dorsiflexion of the ankle for eight days.” The patient participated in a 7-km hiking race for 1.5 hours on September 27, 2020, and began to experience pain and discomfort in front of his right calf half an hour after the race. One hour later, he was admitted to a local hospital and was diagnosed with muscle strain. The patient was administered symptomatic treatment for pain and recommended rest. Pain in the front of the leg worsened when the ankle joint and toe were flexed and extended that night, and the patient developed gross hematuria. Subsequently, the patient remained in bed and was treated with oral pain medication, without significant symptom relief. On October 4, 2020, the patient was admitted to our hospital because of “right calf pain and dorsiflexion of the ankle for eight days.” The outpatient report was “common peroneal nerve entrapment? “ Physical examination on admission: The skin color of the right calf was normal, without obvious swelling, and the tension of the skin and subcutaneous tissue was fine. During deep palpation, the tension of the anterior compartment of the leg was higher, and the intensity of deep tenderness was positive. No obvious abnormalities were observed in the lateral and posterior compartments and the dorsalis pedis artery pulse was not palpable. The back skin of the toe and second toe felt numb, and the ankle and toe could not flexibly flex. The muscle strength was zero grade. On admission, the patient was generally in good condition, with no underlying disease or history of special drug use. Laboratory tests showed a white blood cell count of 10.43 × 10^9^/L (reference value (ref) 4–10 × 10^9^/L) and an absolute neutrophil of 7.91 × 10^9^/L (ref. 2–7 × 10^9^/L), and erythrocyte sedimentation rate was 64 mm/h (ref. 0–15 mm/h) and the C-reactive protein was 130.0 mg/L (ref. 0–8.0 mg/L). The alanine transaminase was 223 U/L (ref. 5–40 U/L) and aspartate aminotransferase was 242 U/L (ref. 8–42 U/L), and creatinine level of 68 µmol/L (ref. 41–110 U/L), blood urea nitrogen was 5.0 mmol/L (ref. 2.9–8.2 mmol/L). After full communication with the patient and his family, a right leg fasciotomy was performed under spinal epidural anesthesia the next day. A longitudinal incision of approximately 20 cm in length was made in the anterior compartment of the leg. After the skin and subcutaneous tissue were cut, the tension of the anterior compartment was very high, and most of the tibialis anterior muscle, extensor digitorum longus, and extensor digitorum longus were necrotic without necrotic borders. The shape of the deep branches of the common peroneal nerve and anterior tibial artery were satisfactory, and the pulse of the anterior tibial artery was restored after adequate decompression (Figs. 1 and 2). The anterior intermuscular septum was incised and the peroneus longus and brevis were normal. Tension of the lateral intermuscular septum was acceptable. The patient’s family strongly requested muscle preservation and refused to clean necrotic muscle. Vacuum sealing drainage (VSD) was used to cover the wound surface (Fig. 3). Postoperative negative pressure drainage was applied to the wound and the pressure was maintained at 6.7–26.7 kpa. Six days after the initial surgery, a second exploratory debridement was performed. No improvement in muscle vascularity was observed during surgery (Fig. 4). After communicating with the patient’s family, necrotic muscle debridement was performed, leaving 1/4 of the well-vascularized muscle remaining after debridement (Fig. 5). VSD closure was again applied to cover the wound surface (Fig. 6). Continuous negative pressure drainage was continued, and the pressure was maintained at 6.7–26.7 kpa. Ten days after the second debridement, the covering foam material was removed, the incision was sutured (Fig. 7), and the patient was immobilized with an ankle neutral brace postoperatively to guide the patient in rehabilitation exercises. The patient was discharged 13 days after the third operation. At the time of discharge, the patient’s liver and kidney functions were normal, the skin of the incision healed well, the blood supply to the lower extremity was normal, and the ankle joint could not be actively dorsiflexed. Laboratory tests showed a white blood cell count of 5.24 × 10^9^/L, an absolute neutrophil of 3.15 × 10^9^/L, an erythrocyte sedimentation rate of 5 mm/h, C-reactive protein of 5.3 mg/L, alanine transaminase of 78 U/L, and aspartate aminotransferase of 37 U/L. At 3-month follow-up after discharge, the patient’s ankle flexor dorsi muscle strength improved to grade 3 (Figs. 8 and 9). At 6-month follow-up, the ankle flexor dorsi muscle strength improved to grade 4 and the patient was able to walk independently. After 1 year of follow-up, the strength of the ankle dorsiflexion muscle improved to grade 5, the patient was able to run and jump freely.

## 3. Discussion

We report a case of acute exertional OCS after short-term, low-impact exercise that was delayed for 8 days and underwent 3 operations. Three months after the operation, the nerve injury and muscle strength significantly improved.

The differential diagnosis of acute nontraumatic leg pain includes vascular, infectious, neurogenic, and metabolic causes.^[6]^ Acute exertional OCS is very rare^[7,8]^ and is characterized by persistent pain that exceeds the expected level of potential injury and requires more analgesia than expected. Delays in the diagnosis and treatment of acute exertional OCS are common but can lead to serious and devastating consequences within hours if not treated in a timely manner.^[9]^ The most common cause of OCS is long bone fracture. Acute exertional cases such as this are rare and have been reported to be associated with hemophilia,^[10]^ disseminated intravascular coagulation,^[11]^ arteritis,^[12]^ and anticoagulation.^[13,14]^ Early diagnosis and treatment of OCS are very important. The typical symptoms and signs of OCS are summarized as “5p” sign: pain, paleness, pulseless, paralysis and paresthesia. Pain is the earliest and most common clinical manifestation. Early paresthesia should be considered an alarm signal resulting from nerve compression. Paralysis accompanied by sensory and motor disturbances occurs as paresthesia progresses. Assessment of chamber pressure is helpful in determining the diagnosis, especially in patients with unresponsive diseases.^[15]^

The exact pathophysiological mechanisms of acute exertional OCS remain unclear.^[16–19]^ During exercise, it has been reported that muscle volume can increase by up to 20% and that hydrostatic pressure in tissues increases due to poor compliance of the muscle compartment.^[18,19]^ In the acute phase, venous and lymphatic return is blocked due to intracellular edema, which in turn leads to tissue damage and muscle necrosis.^[19]^ Myocyte death results in the release of interstitial cell content, accumulation of fluid permeability, and further increase in chamber pressure.^[16]^ In this case, because the patient was heavily obese (BMI: 32, height: 182 cm, weight: 106 kg), the subcutaneous fat layer was thick, the superficial palpation tension was moderate, there was no obvious pressure pain, and the patient was missed by the hospital at the initial diagnosis. However, the deep palpation tension was extremely high and deep tenderness was evident. Remind us that obese patients should be strictly examined to avoid missing diagnoses.

Previous studies have suggested that fasciotomy for decompression may not be beneficial in patients with advanced OCS (> 8 h after onset). The patient was treated surgically after symptoms of deep peroneal nerve injury, and the nerve function recovered gradually. VSD was added to this procedure instead of traditional simple fasciotomy. Continuous negative pressure reduces wound exudation and prevents potential lacuna formation. Keeping the wound closed helped prevent infection and restore the integrity of the skin tissue. VSD materials can achieve thorough wound drainage.^[20,21]^ In addition, VSD treatment can quickly decompress, reduce swelling, improve blood circulation, and disinfect wounds.

Therefore, it is important to accurately identify patients with acute exertional OCS, so that treatment can be initiated in a timely manner.

## 4. Conclusions

We present a rare case of acute exertional OCS of the lower extremity in a young patient. Clinicians should be aware of the signs of acute exertional OCS, as early and appropriate treatment can prevent catastrophic sequelae caused by this rare condition. Compartment syndrome constitutes a surgical emergency. Diagnosis and early treatment are pivotal to avoid amputation, limb dysfunction, kidney failure, and death. OCS decompression must be thorough and usually requires a large incision, resulting in large wounds and excessive exudation. Negative pressure drainage can rapidly reduce compartment pressure. Negative pressure on the wound surface stimulates tissue growth and reduces local toxin absorption. Negative pressure also increases blood circulation around the wound. Overall, the limb swelling and OCS symptoms were reduced. Traditional fasciotomy only has a decompression effect and there is no negative pressure in the osseofascial cavity. Therefore, VSD provide an additional therapeutic effect in patients with OCS by applying negative pressure to the wound.

## Author contributions

**Data curation:** Congcong Wang.

**Methodology:** Jun Wang.

**Project administration:** Jun Wang.

**Resources:** Congcong Wang, Shibo Zhao.

**Supervision:** Shibo Zhao

**Visualization:** Wenjing Song.

**Writing—original draft:** Shiwei Liu.

**Writing—review and editing:** Wenjing Song.
